# Notes and News

**Published:** 1899-07-22

**Authors:** 


### Notes and News.

(Collected and Communicated.)

A serious outbreak of diphtheria is reported from Southgate, which originated in the infant schools.

New York is suffering from a strange epidemic of lockjaw. No less than 25 victims are reported during one fortnight.

The Medical Officer of Health for Spalding, in the Fen country, reports an alarming prevalence of cancer in the district, fatalities from this disease forming a percentage of 11·06 of the total deaths.

An enormous quantity of bad fruit has been condemned this week, a long regiment of dust-vans laden with fruit snatched, as one might say, from the furnace of the jam manufacturers, having been paraded at the Southwark Police-court for the magistrate's inspection.

A curious point was raised in regard to such fruit at the Thames Police-court. The strawberries in question had come from abroad, and having already, owing to their condition, been rejected by the customer, the inspector seized them while in the van in the street. There was plenty of evidence that they were bad, and the owner consented to their destruction. But the magistrate asked for evidence that they were "intended for the food of man" at the time they were seized. This could not be given, as they had already been rejected by the customer, and the magistrate refused the order, and also refused to grant a case. This is a complication.

Paris is to be congratulated, and in view of the great concourse of people of all nations which will next year assemble in Paris, perhaps we may say that the world is to be congratulated, on the completion of the great irrigation works for the utilisation of the sewage of Paris, and for the protection of the Seine from contamination by it. If the upper reaches of the Seine could also be protected from being fouled by the suburban districts and by the towns along its banks, and if Paris could obtain a larger and more reliable supply of drinking water than seems as yet to be available, the health conditions of the city would be still further improved.

Three fresh cases of plague are reported from Alexandria. Up to the present time the mortality has reached 26.

An introductory address at the opening of the winter session at the Owens College will be delivered in the Physiological Theatre on Monday, October 2nd, 1899, at four p.m., by Sir J. Crichton Browne, LL.D., M.D., F.R.S. Members of the medical profession are invited to attend.

A special crusade against infectious diseases appears likely to be commenced in France. For the purpose the Deputy for Roanne in the Loire, Mons. Hudiffred, has started a subscription to establish a fund to provide scientific men with apparatus to pursue research with a view to combat the ravages of infectious disease.

Speaking in the House of Commons last Friday, Mr. Chaplin announced that he had appointed a committee to inquire into the use of preservatives and colouring matters in foods generally. Sir Herbert Maxwell is chairman, and the other members of the committee are Professor Thorpe, Dr. Bulstrode, and Dr. Tunnicliffe. The committee has already met to arrange the course of its inquiries.

Complaints have lately been made by members of the House of Commons as to the nuisance caused by smoke from the pottery works in Lambeth. In answer to these complaints, Mr. Chaplain said the matter was in the hands of the local sanitary authorities, who, he understood, were having a watch kept upon the works in question in regard to the emission of smoke, and would take action whenever any nuisance under the Act could be substantiated.

In connection with the military operations on Salisbury plain complete arrangements have been made to ensure that the men of the Bearer Companies and the Field Hospitals, although they may not be over burdened with actual sick or with really wounded, shall participate to the full in the training which is to be derived from such extensive operations as it is proposed to carry out. Last week a complete medical scheme was gone through as if an action had taken place. A fatigue party of unknown strength was told off to represent the wounded who were distributed over the area of the supposed action, and the medical scheme was to make all medical arrangements suitable to the situation prior to the attack, and remove the wounded from the position during the engagement. Eleven ambulance waggons were utilised, together with 4th Column Field Hospital. The work was carried out most expeditiously, the wounded being picked up, and, after receiving first aid, being removed to the temporary hospital and after that to the stationary hospital at the base, where everything was in readiness to receive them, both in regard to treatment and diet. At the close of the exercises General Rundle complimented the medical staff upon the excellent manner in which all had performed their duties, and said he was much impressed with the quietness and order with which everything had been done. We do not gather that there were umpires present, and no report seems to have been given as to how many of the bearers would have survived had the operations taken place under the conditions of actual warfare—probably very few.

